# Parents’ rearing styles and adolescents’ math achievement: the multiple mediating effect of self-control and math anxiety

**DOI:** 10.3389/fpsyg.2024.1413899

**Published:** 2024-10-16

**Authors:** Yanjiao Wang, Lei Han, Yang Tao, Yanyan Ma

**Affiliations:** ^1^School of Psychology, Inner Mongolia Normal University, Hohhot, China; ^2^Lanzhou Petrochemical University of Vocational Technology, Lanzhou, China; ^3^Lanzhou No.91 Middle School, Lanzhou, China; ^4^Central China Normal University, Wuhan, China

**Keywords:** parents’ rearing styles, self-control, math anxiety, math achievement, adolescent

## Abstract

**Introduction:**

This cross-sectional study examined the mechanisms underlying adolescent math achievement by investigating the relationship between parents’ rearing styles (including different dimensions of rearing style) and adolescent self-control, math anxiety, and math achievement based on the ecological systems theory.

**Method:**

A total of 584 junior high school students (*M* age = 12.52) completed the Parenting Style Questionnaire, Self-control Scale, and Math Anxiety Rating Scale and provided their math test scores.

**Results:**

The rearing styles of both fathers and mothers directly predicted adolescents’ math achievement. Maternal rearing style indirectly predicted adolescents’ math achievement through their self-control and math anxiety; however, the indirect effect of paternal rearing style on adolescents’ math achievement was not significant. After distinguishing the three dimensions of rearing styles, we found that paternal emotional warmth can increase adolescents’ self-control, while maternal emotional warmth can reduce adolescents’ self-control. Further, paternal overprotectiveness can directly and positively predict adolescents’ math achievement, while maternal rejection and overprotectiveness can positively predict adolescents’ math achievement. None of the three dimensions of rearing styles can predict math achievement through adolescents’ self-control; however, they can predict math achievement indirectly through adolescents’ math anxiety and the chain-mediation of adolescents’ self-control and math anxiety.

**Discussion:**

Our results suggest both commonalities and differences in how paternal and maternal rearing styles, along with their three dimensions (emotional warmth, rejection, overprotection), predict adolescent math achievement. These findings highlight the importance of paternal and maternal rearing styles on adolescents’ math achievement and underscore the need to examine them separately to better understand their impact.

## 1 Introduction

Math is a fundamental discipline in the fields of science, technology, and engineering (STEM) and one of the best predictors of success therein (Fong et al., 2021; Le and Robbins, 2016). Math achievement, typically measured by students’ scores in standardized math tests, is a crucial metric for assessing learning outcomes, comprehension, and mastery. It also serves as a significant reference factor for students’ academic advancement and career development (Fong et al., 2021; St Omer and Chen, 2023). Adolescence is a critical period in children’s development, during which math achievement is closely linked to negative emotions such as academic boredom (Borgonovi et al., 2023), which further influence children’s future career choices and developmental trajectories (Toh and Watt, 2022). There are at least two theories that can explain the factors that influence adolescents’ math achievement—ecological systems and social cognitive theories. Ecological systems theory suggests that children’s math achievement is affected by both individual and environmental factors (Bronfenbrenner, 1979). Similarly, social cognitive theory holds that environmental and children’s individual factors will have a decisive impact on their math achievement and other accomplishments (Bandura, 1997; Zimmerman, 1989). Based on this, previous studies have found that parents’ rearing styles, as an important environmental factor, and adolescents’ self-control and math anxiety, as important individual factors, all play an important role in adolescents’ math achievement (Bardach et al., 2023; Barroso et al., 2021; Pinquart, 2015). However, previous studies have not revealed the complex nature of this relationship. Thus, this study aimed to extensively investigate the relationship between parents’ rearing styles and adolescents’ self-control, math anxiety, and math achievement from the perspective of both environmental and individual factors. This could help enhance the understanding of the underlying mechanisms affecting adolescents’ math achievement and provide guidance for improvement.

### 1.1 Parents’ rearing styles and adolescents’ math achievement

As the primary context in which children first interact with the outside world, the family is the most direct micro-environment affecting children’s development (Underdown, 2007). Within this micro-environment, parents’ rearing styles are closely related to adolescents’ math achievement (Ogg and Anthony, 2020). Parents’ rearing styles encompass both the attitudes conveyed by parents and the emotional atmosphere created by their behavior and can be categorized as positive or negative. Positive rearing styles are characterized by emotional warmth and care, an emphasis on communication and understanding, and the encouragement of autonomy and independence. Conversely, negative rearing styles are marked by a lack of emotional communication, excessive severity, control, and rejection (Li et al., 2019; Ogg and Anthony, 2020). According to the autonomy-supportive parenting and distance-conflicted family theories (Guo et al., 2021), affective, encouraging, and related parenting styles are characterized by support and autonomy, whereas rejection- and control-based parenting styles are characterized by distance and conflict. The parenting style based on supporting autonomy is more inclusive and creates a more relaxed and pleasant atmosphere at home, making it easier to cultivate children to produce positive results in school and other aspects. Contrastingly, the parenting style based on distance and conflict may cause tension and exert pressure on children, which may hinder their academic development (Guo et al., 2021). Regarding children’s math achievement, empirical studies have found that parenting styles based on autonomy and support features, such as the emotional warmth style, are conducive to the improvement of adolescents’ math performance, while those based on rejection are linked to a reduction in adolescents’ math achievement (Retanal et al., 2021). This reveals that different parenting styles have varying effects on adolescents’ math achievement. To elucidate these varying effects and comprehensively explore the relationship between parents’ rearing styles and adolescents’ math achievement, we subdivided the specific dimensions of different rearing styles.

Most studies have only focused on the combined influence of parents (i.e., fathers and mothers) (Wang and Fletcher, 2015). Recent research, including the male breadwinner-female housewife model, has suggested significant differences in paternal and maternal rearing styles owing to gender, roles, and division of labor between parents (Du et al., 2021; Waismel-Manor and Levanon, 2024). Moreover, this difference leads to variations in the effects of paternal and maternal rearing styles on adolescents’ cognition and behavior (Moilanen et al., 2014). Therefore, by considering paternal and maternal rearing styles separately, we can have a clear understanding of the specific effect of parents’ rearing styles on adolescents’ math achievement, which would enable more targeted prevention and intervention efforts. Thus, by distinguishing the rearing styles of fathers and mothers, this study examines the relationship between the sub-dimensions of parental rearing and adolescents’ math achievement.

### 1.2 Relationship between parents’ rearing styles and adolescents’ self-control and math achievement

Self-control is a crucial psychological function that allows individuals to voluntarily regulate unwarranted thoughts, emotions, and behaviors in alignment with societal norms to support the achievement of long-term goals (Li et al., 2019; Muraven and Baumeister, 2000). Previous studies have found that effective self-control can improve adolescents’ social adaptability and reduce their risk of substance use, emotional problems, and aggressive behavior (Li et al., 2019; Li et al., 2023a; Rodríguez-Ruiz et al., 2023; Tangney et al., 2004), as well as alleviate family conflict and academic burnout in adolescents (Luo et al., 2020). Additionally, self-control can improve academic performance, specifically in math (Duckworth et al., 2019). Empirical studies have shown that self-control is positively correlated with math achievement (Cleary and Chen, 2009; Cleary and Kitsantas, 2017; Dent and Koenka, 2015). The self-control strength model (Muraven, 2010; Muraven and Baumeister, 2000), self-determination theory (Husain, 2023; Peterson et al., 2020; Wang et al., 2022), social cognitive theory (Martin, 2004; Zimmerman, 1989), and other theories support the view that self-control is closely related to academic achievement in subjects such as math. For example, social cognitive theory suggests that students with strong self-control often exhibit higher self-efficacy, which boosts their confidence in completing tasks, increases their willingness to put effort into and persist in mathematical learning, and ultimately enhances their chances of achieving good results in math (Martin, 2004; Zimmerman, 1989).

The ecological systems theory posits that parents’ rearing styles constitute a crucial component of the family environment. Parents’ rearing styles significantly guide and shape the development of adolescents’ self-control (Li et al., 2019; Pallini et al., 2018; Yang et al., 2023) and can indirectly do so through factors such as family atmosphere and parent–child relationships (Moilanen et al., 2014; Mun et al., 2018). For example, emotional warmth and other positive rearing styles, provide adolescents with appropriate care and support and reasonably limit and guide their behavior, enabling them to achieve balanced development in self-cognition, emotional regulation, and behavior control. In contrast, rejection-based and other negative rearing styles, are more likely to cause parents to ignore the needs of adolescents, provide insufficient guidance and education, and lack necessary care and support. As a result, adolescents may struggle to develop effective self-control (Abedini et al., 2012; Li et al., 2014; Moilanen et al., 2014). Similar findings have been observed across different cultural backgrounds and age groups (Finkenauer et al., 2005; Li et al., 2023b; Yang et al., 2023), suggesting that parents’ rearing styles may affect adolescents’ self-control, with parents and their specific parenting styles potentially having varying effects on self-control. In addition, considering the relationship between parents’ rearing styles (and their differences) and adolescents’ math achievement, as well as adolescents’ self-control and math achievement, this study proposes the following hypotheses:

*H1:* Adolescents’ self-control mediates the relationship between parents’ rearing styles and adolescents’ math achievement. However, this mediating role differs between parents (i.e., paternal or maternal rearing styles).

*H2:* The above mediating effect is also related to the specific rearing style adopted by fathers or mothers (for example, whether it is emotional warmth or rejection).

### 1.3 Relationship between parents’ rearing styles and adolescents’ self-control, math anxiety, and math achievement

How does self-control impact adolescents’ math achievement? First, multiple theories have explained the relationship between self-control and emotions (Burt, 2020; King and Gaerlan, 2014). For instance, according to the cognitive theory of emotion, individual cognition can affect emotion and is the key factor determining the nature of emotion (Brewin, 1996; Oatley and Johnson-Laird, 2014). Compared to adolescents with effective self-control, those with poor self-control are more likely to experience negative emotions such as math anxiety, which can be exacerbated by reduced self-efficacy (Jain and Dowson, 2009). In addition, according to the motivational theory of emotion, children’s math anxiety can reduce their interest and motivation in learning math by negatively impacting their enthusiasm and initiative. This lack of emotion and motivation may result in insufficient investment in math learning, ultimately leading to reduced math achievement (John et al., 2020; Wang et al., 2015). Research based on behavioral tests has confirmed that individuals with increased math anxiety tend to avoid math problems (Choe et al., 2019); this, in turn, hinders math achievement (Wang et al., 2021). Therefore, we formulate the following hypothesis:

*H3:* Adolescents’ math anxiety plays a mediating role in the relationship between self-control and math achievement.

In addition, previous research has indicated that parents’ rearing styles may affect adolescents’ math anxiety. For example, Macmull and Ashkenazi (2019) have found that controlling and punishing rearing styles are associated with adolescents’ high math anxiety, and the more supportive and encouraging rearing styles lower adolescents’ math anxiety. Based on the relationship between parents’ rearing styles and adolescents’ math achievement, and the relationship between adolescents’ math anxiety and math achievement, we posit the following hypotheses:

*H4:* Adolescents’ math anxiety mediates the relationship between the specific rearing style adopted by parents (e.g., emotional warmth) and adolescents’ math achievement. Moreover, the mediating effect of math anxiety between paternal rearing style and adolescents’ math achievement is different from that between maternal rearing style and adolescents’ math achievement because of the possible differences between paternal and maternal parenting styles.

*H5:* Adolescents’ self-control and math anxiety play a chain mediating role between parents’ rearing styles and adolescents’ math achievement. This chain mediation may be related to each parent (e.g., the paternal versus maternal rearing styles).

*H6:* The chain mediating role of adolescents’ self-control and math anxiety in the relationship between different dimensions of parents’ rearing styles and adolescents’ math achievement varies.

This study draws on ecological systems theory and considers both environmental and individual factors affecting adolescents. From the perspectives of both fathers and mothers, this study explores the relationships between paternal and maternal rearing styles (including the emotional warmth, rejection, and overprotection dimensions) and adolescents’ self-control, math anxiety, and math achievement.

## 2 Materials and methods

### 2.1 Participants

This study employed a convenience sampling method to conduct a collective questionnaire survey in December 2023 with students from two full-time middle schools in Lanzhou, China. A total of 600 questionnaires were distributed. Sixteen participants were excluded due to incomplete or unserious responses, resulting in 584 valid questionnaires with an effective response rate of 97.33%. The sample consisted of 278 male (47.60%) and 306 female (52.40%) students, ranging in age from 12 to 15 years, with an average age of 12 years (*M* = 12.52, *SD* = 1.04). The studies involving human participants were reviewed and approved by the Scientific Research Ethics Committee of the School of Psychology of Northwest Normal University (Approval No. 2023101). The study was conducted in the schools after verbal informed consent had been provided by the heads of middle schools and the children’s parents.

### 2.2 Measures

#### 2.2.1 Parents’ rearing styles

This study used the Chinese version of the parental bonding instrument (PBI), which was translated and revised by Jiang et al. (2010). The original scale is divided into two parts for paternal and maternal rearing styles, with a total of 42 items. The scale comprises three dimensions: rejection, emotional warmth, and overprotection, which include six, seven, and eight items, respectively. All the items are scored on a five-point Likert scale, with higher subscale scores indicating a stronger tendency toward a particular rearing style. The average scores for the three dimensions of emotional warmth, rejection, and overprotection were calculated separately for fathers and mothers. The emotional warmth dimension was used to assess positive rearing styles, whereas the rejection and overprotection dimensions were used to assess negative rearing styles (Li, 2018). The Cronbach’s alpha coefficients for the three dimensions for the fathers and mothers ranged from 0.72 to 0.85, indicating good reliability.

#### 2.2.2 Self-control scale

The study employed the Chinese version of the self-control dual-system scale (Xie et al., 2014). This scale comprises 21 items and is divided into two subscales: impulsive system and control system. The impulsive system includes three dimensions: impulsivity, distractibility, and low delay of gratification. The control system encompasses two dimensions: problem-solving and future time perspective. The scale uses a five-point Likert scale (1 = “strongly disagree”; 5 = “strongly agree”). In this study, Cronbach’s alpha for the overall scale was 0.71.

#### 2.2.3 Math anxiety scale

The study employed the Chinese version of the Mathematics Anxiety Rating Scale for children, which was originally developed by Suinn and Winston (2003). The Chinese version is considered an effective tool for assessing math anxiety among Chinese adolescents (Wu, 2014). The scale comprises 27 items and uses a five-point Likert scale (1 = “not anxious at all”; 5 = “extremely anxious”). The adolescents reported their anxiety levels, with higher scores indicating higher levels of anxiety. In this study, Cronbach’s alpha for the overall scale is 0.87.

#### 2.2.4 Math achievement

Data on participants’ math achievement were obtained from school records, specifically the average scores of their two most recent major math examinations. Previous studies have found that performance in math courses effectively reflects academic achievement in Chinese children (Chen et al., 1997; Ding et al., 2012). During data analysis, we converted the math scores of all participants in this study. The resulting Z-scores were used as the final metric for analyzing math achievement.

### 2.3 Procedure

This study targeted first- and second-year junior high school students and conducted assessments in stages. Before administering the tests, math or homeroom teachers were requested to exclude children with sensory deficits or intellectual disabilities. Subsequently, the tests were conducted in classrooms, with each class assigned two rigorously trained psychology professionals with extensive experience in administering psychological tests as the primary testers. The testers collected data on the adolescent parents’ parenting styles, the adolescents’ self-control, math anxiety, and math scores on the last two major examinations. Prior to answering the questionnaires, the primary testers provided detailed instructions to the participants and answered their questions. The instructions emphasized the significance of the survey and the confidentiality of the results and required participants to respond independently based on their actual situations. After the administration, the questionnaires were collected by the primary testers. Questionnaire administration and score collection were conducted with the consent of both the students and the schools. The participants were given 25 min to complete the questionnaires and received a small gift upon completion.

### 2.4 Analysis

SPSS 26.0 was used for the three-stage statistical analysis. First, we tested the skewness of each variable and performed a correlation analysis. Second, after standardizing all variables and evaluating multicollinearity by testing the variance inflation factor (VIF), a mediating analysis was performed to test: (1) Whether paternal and maternal rearing styles have the same predictive effect on adolescents’ math achievement and if there are any differences of the predictive effect of the three different rearing styles on adolescents’ math achievement; (2) Whether adolescents’ self-control plays a mediating role in the relationship between paternal and maternal rearing styles and adolescents’ math achievement, and whether it plays the same role between the three different rearing styles and adolescents’ math achievement; (3) Whether adolescents’ math anxiety plays a mediating role in the relationship between their self-control and math achievement; and (4) Whether adolescents’ self-control and math anxiety play a chain-mediating role in the relationship between paternal and maternal rearing styles and math achievement, and whether the three different rearing styles play the same role in the chain-mediating relationship.

For the above analyses, we established two models with adolescents’ math achievement as the dependent variable. In the first model, paternal (maternal) rearing style was the independent variable, and self-control and math anxiety were the mediating variables. In the second model, the three dimensions of rearing style (emotional warmth, rejection, overprotection) were the independent variables, and self-control and math anxiety were mediating variables.

Finally, we further verified the mediation effect of the above model. The PROCESS macro in SPSS (Hayes, 2013) was used to calculate the predictive effects of independent variables on dependent variables in each model. This included both the direct effects of independent variables on mediating variables and the direct effects of mediating variables on dependent variables, as well as the indirect effects of independent variables on dependent variables through mediating variables. The indirect effects of the size of the bias-corrected bootstrapped confidence interval (95% CI) (10,000 samples) without zero indicated that the mediation effect was significant; otherwise, it was deemed not significant. The results of the mediation analysis were reported after standardization.

## 3 Results

### 3.1 Descriptive statistics

The mean, standard deviation, and correlation matrix of paternal and maternal rearing styles and adolescents’ self-control, math anxiety, and math achievement are shown in Table 1. After distinguishing the three dimensions of rearing styles, the mean, standard deviation, and correlation matrix of variables are shown in Table 2. The skewness analysis shows that the skewness values of each variable were between −1 and 1, indicating that there was no serious skewness distribution. Moreover, after normalizing all variables, the variance inflation factor was less than 10, indicating no multicollinearity issues.

**Table 1:** Correlation analysis of the relationship between parents’ rearing styles and adolescent’ self-control, math anxiety and math achievement.

Variable	*M ± SD*	1	2	3	4
1 Parents’ rearing styles	57.22 *±* 8.95	-	0.08^*^	0.06	0.09^*^
2 Self-control	57.53 *±* 9.81	0.13^**^	-	0.23^**^	−0.06
3 Math anxiety	75.93 *±* 20.09	0.11^**^	0.23^**^	-	−0.41^**^
4 Math achievement	71.13 *±* 28.99	0.11^**^	−0.06	−0.41^**^	-
*M ± SD*		58.48 *±* 9.21	Same above	Same above	Same above

Data of father on the diagonal line, data of mother on the diagonal line, *p < 0.05; **p < 0.01; ***p < 0.001.

**Table 2:** Correlation analysis of the three dimensions of rearing style and adolescent’ self-control, math anxiety and math achievement.

Variable	*M ± SD*	1	2	3	4	5	6
1. Emotional warmth	24.98 *±* 6.81	-	−0.41^**^	−0.35^**^	−0.11^**^	−0.30^**^	0.18^**^
2. Rejection	13.66 *±* 5.03	−0.40^**^	–	0.68^**^	0.18^**^	0.28^**^	−0.06
3. Overprotection	18.59 *±* 5.14	−0.34^**^	0.72^**^	–	0.12^**^	0.24^**^	−0.02
4. Self-control	57.53 *±* 9.81	−0.09^*^	0.19^**^	0.16^**^	–	0.23^**^	−0.06
5. Math anxiety	75.93 *±* 20.09	−0.23^**^	0.27^**^	0.22^**^	0.23^**^	–	−0.41^**^
6. Math achievement	71.13 *±* 28.99	0.15^**^	−0.03	0.04	−0.06	−0.41^**^	-
*M ± SD*		25.81 *±* 6.51	13.60 *±* 5.02	19.07 *±* 5.33	57.53 *±* 9.81	75.93 *±* 20.09	71.13 *±* 28.99

Data of father on the diagonal line, data of mother below the diagonal line. *p < 0.05; **p < 0.01; ***p < 0.001.

### 3.2 Mediating analysis of rearing styles, self-control, math anxiety, and math achievement

**Figure 1:**
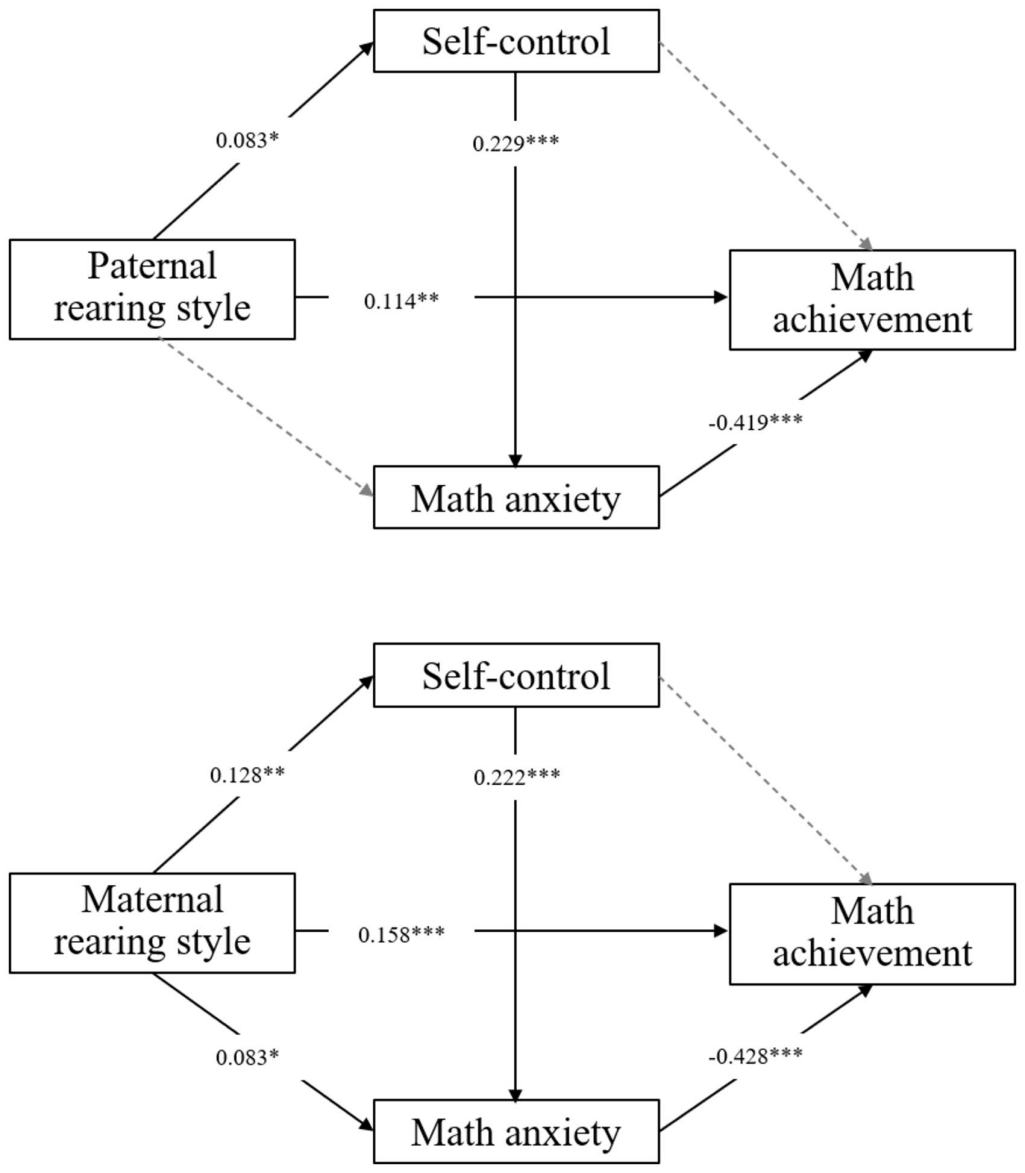
Relationship between paternal and maternal rearing styles and adolescents’ self-control, math anxiety, and math achievement.

The mediation analysis revealed that paternal rearing style positively predicted adolescents’ self-control (*β* = 0.083, *p* = 0.044) (Figure 1). Adolescents’ self-control positively predicted their math anxiety (*β* = 0.229, *p* < 0.001). Paternal rearing style positively predicted adolescents’ math achievement (*β* = 0.114, *p* = 0.003). Adolescents’ math anxiety negatively predicted their math achievement (*β* = −0.419, *p* < 0.001). Paternal rearing style did not significantly predict adolescents’ math anxiety (*β* = 0.041, *p* = 0.310), and adolescents’ self-control did not significantly predict their math achievement (*β* = 0.027, *p* = 0.488). Moreover, maternal rearing style positively predicted adolescents’ self-control (*β* = 0.128, *p* = 0.002). Both maternal rearing style (*β* = 0.083, *p* = 0.041) and adolescents’ self-control (*β* = 0.222, *p* < 0.001) positively predicted adolescents’ math anxiety. Maternal rearing style positively predicted adolescents’ math achievement (*β* = 0.158, *p* < 0.000). Adolescents’ math anxiety negatively predicted their math achievement (*β* = −0.428, *p* < 0.001), while self-control had no significant predictive effect on math achievement (*β* = 0.018, *p* = 0.637). In addition, the mediation effect analysis found that the mediation effect of paternal rearing style on the three paths of math achievement was not significant (Table 3). There was a significant chain-mediated effect of adolescents’ self-control and math anxiety on the relationship between maternal rearing style and adolescents’ math achievement [95CI (−0.028, 0.001), without passing 0].

**Table 3:** The mediating effect of self-control and math anxiety on the relationship between parents’ rearing styles and adolescents’ math achievement.

Pathways	Paternal			Maternal		
	Indirect effects	*SE*	95%CI	Indirect effects	*SE*	95%CI
RS → SC → MAc	0.002	0.005	−0.006, 0.016	0.002	0.007	−0.009, 0.018
RS → MAn → MAc	−0.017	0.020	−0.056, 0.023	−0.036	0.021	−0.078, 0.006
RS → SC → MAn → MAc	−0.008	0.007	−0.023, 0.004	−0.012	0.007	−0.028, −0.001
Total indirect effect of RS → MAc	−0.023	0.020	−0.061, 0.016	−0.045	0.021	−0.088, −0.005

RS, rearing styles; SC, self-control; MAc, math achievement; MAn, math anxiety.

To investigate the relationship between the three dimensions of rearing style and adolescents’ self-control, math anxiety, and math achievement, we explored these connections from both the perspective of fathers and mothers. The results show that there are both similarities and differences between the three dimensions of paternal and maternal rearing styles and adolescents’ self-control, math anxiety, and math achievement (Table 4). Specifically, paternal emotional warmth positively predicted adolescents’ self-control, while maternal emotional warmth negatively predicted adolescents’ self-control. Only paternal overprotection positively predicted adolescents’ math achievement, while both maternal rejection and overprotection positively predicted adolescents’ math achievement. The similarity is that both paternal and maternal rejection and overprotection positively predicted adolescents’ self-control. Both paternal and maternal emotional warmth negatively predicted adolescents’ math anxiety, but they had no significant predictive effect on adolescents’ math achievement. Rejection and overprotection by both fathers and mothers were positive predictors of adolescents’ math anxiety. In addition, adolescents’ self-control consistently positively predicted their math anxiety, and adolescents’ math anxiety consistently negatively predicted their math achievement.

**Table 4:** Standard coefficients between the three dimensions of paternal and maternal rearing styles and self-control, math anxiety, and math achievement.

Pathways	Paternal	Maternal
Emotional warmth → Self-control	0.112^**^	−0.093^*^
Rejection → Self-control	0.183^***^	0.186^***^
Overprotection → Self-control	0.115^**^	0.159^**^
Emotional warmth → Math anxiety	−0.281^***^	−0.210^***^
Rejection → Math anxiety	0.240^***^	0.231^***^
Overprotection → Math anxiety	0.215^***^	0.191^***^
Emotional warmth → Math achievement	0.060	0.060
Rejection → Math achievement	0.025	0.079^*^
Overprotection → Math achievement	0.083^*^	0.138^**^
Self-control→ Math achievement	0.201^***^/0.188^***^/0.207^***^	0.213^***^/0.189^***^/0.202^***^
Math anxiety → Math achievement	−0.396^***^/−0.427^***^/−0.433^***^	−0.401^***^/−0.433^***^/−0.441^***^

RS, rearing styles; SC, self-control; MAc, math achievement; MAn, math anxiety.

The three data points between self-control and math anxiety, and math anxiety and math achievement are standardized regression coefficients under three different parenting styles.

The results of the mediation effect test show that the mediating effects of adolescents’ self-control between paternal (maternal) emotional warmth and math achievement, paternal (maternal) rejection and math achievement, and paternal (maternal) overprotection and math achievement were not significant (Table 5). However, adolescents’ math anxiety had a significant mediating effect between paternal (maternal) emotional warmth and adolescents’ math achievement, paternal (maternal) rejection and math achievement, and paternal (maternal) overprotection and math achievement. Moreover, adolescents’ self-control and math anxiety had significant chain-mediated effects in the chain between paternal (maternal) emotional warmth and math achievement, paternal (maternal) rejection and math achievement, as well as paternal (maternal) overprotection and math achievement.

**Table 5:** The mediating effect of self-control and math anxiety on the three dimensions of parents’ rearing styles and adolescents’ math achievement.

Paths	Paternal			Maternal		
	Indirect effects	*SE*	95%CI	Indirect effects	*SE*	95%CI
EW → SC → MAc	−0.004	0.006	−0.018, 0.005	−0.004	0.005	−0.016, 0.005
EW → MAn → MAc	0.111	0.021	0.073, 0.154	0.084	0.020	0.048, 0.125
EW → SC → MAn → MAc	0.009	0.005	0.002, 0.019	0.008	0.005	0.001, 0.018
Total indirect effect of EW → MAc	0.116	0.021	0.077, 0.159	0.089	0.020	0.052, 0.131
RE → SC → MAc	0.005	0.009	−0.010, 0.026	0.005	0.009	−0.011, 0.025
RE → MAn → MAc	−0.102	0.021	−0.145, −0.062	−0.099	0.021	−0.142, −0.060
RE → SC → MAn → MA	−0.015	0.006	−0.029, −0.005	−0.015	0.007	−0.030, −0.005
Total indirect effect of RE → MAc	−0.112	0.022	−0.155, −0.070	−0.110	0.021	−0.153, −0.070
OV → SC → MAc	0.003	0.006	−0.006, 0.019	0.003	0.008	−0.010, 0.021
OV → MAn → MAc	−0.093	0.020	−0.134, −0.054	−0.084	0.021	−0.126, −0.044
OV → SC → MAn → MA	−0.010	0.006	−0.024, −0.001	−0.014	0.006	−0.029, −0.004
Total indirect effect of OV → MAc	−0.100	0.021	−0.143, −0.060	−0.095	0.022	−0.138, −0.053

EW, emotional warmth; RE, rejection; OV, overprotection; SE, standard error of the coefficient.

## 4 Discussion

Based on the ecosystem theory, this study examined the relationship between parents’ rearing style and adolescents’ self-control, math anxiety, and math achievement from the perspective of both environmental and individual factors influencing adolescents’ math achievement. To our knowledge, this is the first study to examine separately the relationship between paternal and maternal rearing styles (including the three dimensions of rearing styles) and adolescent self-control, math anxiety, and math achievement. We found that both paternal and maternal rearing styles were positive predictors of adolescents’ math achievement. In addition to the significant chain-mediated effect of adolescents’ self-control and math anxiety between maternal rearing style and adolescents’ math achievement, the (chain-mediated) effect of adolescents’ self-control and math anxiety between paternal rearing style and math achievement, and the mediating effect of adolescents’ self-control (math anxiety) between maternal rearing style and adolescents’ math achievement were not significant. After distinguishing the rearing styles’ three dimensions, this study further found that adolescents’ math anxiety always plays a mediating role between the three dimensions of paternal and maternal rearing styles and math achievement, adolescents’ self-control and math anxiety play a chain mediating role between the three dimensions of paternal and maternal rearing styles and math achievement. The mediating effect of adolescents’ self-control on the three dimensions of paternal and maternal rearing styles and math achievement was not significant. However, the specific parenting styles adopted by fathers and mothers differ in their predictive effects on adolescents’ math achievement. The above results are not entirely consistent with our expectations. The results suggest that although paternal and maternal rearing styles both directly predict adolescents’ math achievement, their indirect prediction effects on adolescents’ math achievement vary. Moreover, there are similarities and differences between the three dimensions of the paternal and maternal rearing styles in predicting adolescents’ math achievement through math anxiety and the mediating role of self-control and math anxiety.

In this study, we found that both paternal and maternal rearing styles positively predicted adolescents’ math achievement, which is consistent with ecological theory (Bronfenbrenner, 1979). This study further found that paternal and maternal emotional warmth, rejection, and overprotection predict adolescent math achievement through math anxiety and the chain mediation of self-control and math anxiety, enriching the ecosystem and social cognition theories. These results are also consistent with those of research based on the relationship between parents’ (paternal and maternal) rearing styles and adolescents’ math anxiety (Jin et al., 2024; Wang et al., 2023), as well as previous meta-analysis and recent follow-up studies that found a close relationship between adolescent math anxiety and math achievement (Barroso et al., 2021; St Omer and Chen, 2023). Some studies have found that both parental emotional warmth and rejection can predict adolescent depression and other emotions (Li et al., 2024; Wang et al., 2023). Moreover, parental emotional warmth and rejection are associated with adolescent personality development and creativity (Guo et al., 2021). These results indicate that less emotional warmth of both fathers and mothers will increase adolescents’ math anxiety, which is not conducive to the improvement of math achievement. Further, the higher the level of overprotection and rejection, the higher the adolescents’ math anxiety, which is also not conducive to the improvement of adolescents’ math achievement. However, both fathers and mothers should pay attention to their rearing styles as they are important for adolescents’ math achievement and healthy growth.

While both paternal and maternal rearing styles positively predict math achievement, the predictive effects of paternal and maternal rearing styles on adolescent math achievement vary. This variation is mainly reflected in the following three aspects. First, the indirect predictors of math achievement differ between paternal and maternal rearing styles. The paternal rearing style does not predict adolescent math achievement through self-control, math anxiety, or the chain mediation between self-control and math anxiety; however, the maternal rearing style does predict math achievement through the chain mediation between self-control and math anxiety. This may be related to the different roles and division of labor between fathers and mothers in housework. For example, according to the male breadwinner-female housewife model, in most Chinese families, fathers are more focused on working or earning a living, while mothers are more focused on family and childcare (Waismel-Manor and Levanon, 2024). Therefore, maternal rearing styles predict adolescent math achievement through the indirect effect of adolescent self-control and math anxiety, indicating that the indirect prediction effects of paternal and maternal rearing styles differ. More obvious maternal rearing styles lead to increased adolescent self-control. However, this may increase adolescent math anxiety, which, in turn, can negatively affect adolescent math achievement. Therefore, this result highlights how maternal rearing styles play a role in adolescents’ math achievement.

Second, we found that different dimensions of paternal and maternal rearing styles variably directly predicted adolescent math achievement. Only the overprotection of fathers can directly and positively predict adolescents’ math achievement, while maternal rejection and overprotection can positively predict adolescents’ math achievement. This is inconsistent with the autonomy-supportive and distance-conflict theory (Guo et al., 2021). Such differences may be related to factors such as family education and children’s cognition caused by cultural differences between China and the West (Li et al., 2019). In addition, the differences between paternal and maternal rearing styles and the three dimensions of rearing styles are considered (Li et al., 2024). Our results suggest that, on the one hand, fathers should devote more time to adolescents’ daily education and care to balance with mothers. On the other hand, mothers, who often accompany and care for adolescents at home, should appropriately let go and let adolescents make their own decisions to cultivate their sense of responsibility, making adolescents more active and tenacious in facing problems, such as those in math. Furthermore, if necessary, educational supervision can be strengthened to help adolescents improve their math and other achievements.

Third, the three dimensions of paternal and maternal rearing styles vary in predicting adolescents’ self-control. Maternal emotional warmth reduces adolescent self-control, while paternal emotional warmth increases adolescent self-control. This is consistent with previous studies and corresponding theoretical views (Cullen et al., 2008). According to the ecological theory, environmental factors (such as parents’ rearing style) play crucial roles in the self-control of individuals (Bronfenbrenner, 1979; Li et al., 2019). In addition, according to autonomy-supportive parenting theory (Guo et al., 2021), too much or too little parental warmth and support may be detrimental to the development of various abilities of children, such as self-control (Retanal et al., 2021). Therefore, the findings of this study indicate that mothers should be cautious when adopting emotional warmth rearing style, as excessive levels of emotional warmth may not be conducive to adolescents’ self-control.

In addition, this study found that self-control consistently positively predicts math anxiety in adolescents. This contradicts previous research findings based on adolescent participants. For instance, Tevfik (2015) has found a significant negative relationship between adolescents’ self-control and math anxiety. Our results may align with Kremen and Block’s (1998) explanation, which suggests that the benefits of self-control follow a curved pattern. Insufficient control (defined as low self-control) predisposes individuals to undesirable outcomes such as anxiety and antisocial behavior. However, excessive control (defined as high self-control) may suppress spontaneity, creativity, and joy in learning and other areas of life. They further propose that insufficient control may be associated with behavioral issues such as criminality and aggression, while excessive control may be linked to emotional problems such as anxiety and depression (Finkenauer et al., 2005). Our findings suggest that excessive self-control among adolescents may increase math anxiety.

We further found a significant negative correlation between math anxiety and adolescents’ math achievement. This differs from a recent study conducted in a Chinese cultural context (St Omer and Chen, 2023). In their one-year longitudinal study involving Taiwanese adolescents, St Omer and Chen (2023) found a significant positive relationship between math anxiety and math achievement. This discrepant result could stem from differences in the participant setting, sample sizes, and measurement tools for math anxiety between the two studies (Namkung et al., 2019). Specifically, we selected 584 children from economically and relatively underdeveloped northwestern regions of mainland China, while St Omer and Chen (2023) included 335 children from economically developed Taiwan. Additionally, our study employed a math anxiety scale revised by Chinese scholars that has been considered appropriate for measuring math anxiety among Chinese children, whereas St Omer and Chen (2023) used a more widely applicable math anxiety scale. Our findings indicate that within the same cultural background, the relationship between adolescents’ math anxiety and math achievement can vary. In mainland China, adolescents’ math anxiety is detrimental to improving their math achievement.

## 5 Limitations and implications

This study has several limitations. First, it is a cross-sectional study, which precludes the investigation of causal relationships between variables. Future research could employ longitudinal studies to examine bidirectional and causal relationships between variables. Second, the methodology of this study relies on self-reports from adolescents to measure perceived parenting styles, rather than actual parenting practices. Future studies could utilize multi-informant assessments to gain a more comprehensive understanding of the role of parenting styles. Last, the study employed a convenience sampling method, recruiting first- and second-year junior high school students as participants, which may limit the sample’s representativeness. Future research should employ more systematic sampling methods to include adolescents and children of various age groups, enhancing the representativeness of the sample.

However, this study holds significant theoretical and practical implications. Our findings reveal that parenting styles, especially maternal rearing styles, have a direct or indirect predictive effect on adolescents’ math achievement. Further, we distinguished the three dimensions of rearing style and found both similarities and differences in the influence thereof on adolescents’ math achievement. This has enriched the ecosystem theory research on the relationship between parents’ parenting style and adolescents’ math achievement. Second, to comprehensively analyze the formation mechanism of adolescents’ math achievement, our study focused on environmental factors (rearing style) and individual adolescent factors (self-control and math anxiety). This perspective and the corresponding results expand the application of social cognitive, emotional motivation and other related theories in the field of education and provide a reference for follow-up research regarding adolescent math achievement. Finally, this study found that math anxiety is a stable mediating variable between the three dimensions of parents’ rearing styles and adolescents’ math achievement, which provides an important basis for understanding the role of math anxiety in the formation of academic achievement.

This study has practical implications for alleviating adolescents’ math anxiety and improving their math achievement. We found that paternal and maternal emotional warmth negatively predicts adolescents’ math anxiety, parental rejection and overprotection positively predict adolescents’ math anxiety, the three dimensions of paternal and maternal rearing styles predict adolescents’ self-control and math anxiety, and the chain mediators of math anxiety and self-control predict math achievement. These findings provide guidance for family education as follows: First, adolescence is a critical period for the development of math anxiety (Wang et al., 2020). When educating children, both parents should pay attention to positive rearing styles such as warmth and support, and avoid negative rearing styles such as rejection and punishment to reduce adolescents’ math anxiety and improve their math performance. Second, the emotional communication between father/mother and adolescents should be controlled so that adolescents can feel paternal and maternal warmth. At the same time, parents should appropriately ‘let go’ to exercise adolescents’ sense of autonomy and responsibility, promote the balanced development of adolescents’ mental health, and help adolescents improve their math performance.

## 6 Conclusion

Both paternal and maternal rearing styles can significantly predict adolescents’ math achievement. Moreover, maternal rearing styles can indirectly predict adolescents’ math achievement through self-control and math anxiety. Furthermore, paternal and maternal emotional warmth can reduce adolescents’ math anxiety, and thus improve adolescents’ math achievement. While parental rejection and over-protection may appear to enhance adolescents’ self-control, they actually hinder the improvement of adolescents’ math achievement by increasing math anxiety. Our results suggest the importance of parental, especially maternal, rearing styles to adolescent math achievement and the difference between paternal and maternal rearing styles in predicting adolescent math achievement. Parents should adopt more warm, positive, and supportive rearing styles and less rejection, punishment, and other negative rearing styles; furthermore, parents should grasp a certain degree of such styles to increase adolescents’ self-control, reduce their math anxiety, and help them improve their math performance.

## Data Availability

The datasets presented in this article are not readily available due to the sensitivity of the data: adolescent math achievement. Requests to access the datasets should be directed to the corresponding author.
